# New Method of Addressing Journal

**Published:** 1916-05

**Authors:** 


### New Method of Addressing Journal.

Beginning with the April issue, we began addresing the Journal by a stencil system. Owing to unavoidable delay in securing the machine after the stencils were made, it was necessary to use an old machine so that the printing was poor. Even so, time was saved as compared with other methods which have been used in the past. Beginning with the present issue, subscribers are requested to notify us of errors in address of any kind, although we may not be able to correct minor errors at once. Individuals and societies having regular mailing lists are referred to our advertising columns.

